# Using patient reported outcome measures in health services: A qualitative study on including people with low literacy skills and learning disabilities

**DOI:** 10.1186/1472-6963-12-431

**Published:** 2012-11-26

**Authors:** Deepa Jahagirdar, Thilo Kroll, Karen Ritchie, Sally Wyke

**Affiliations:** 1 Institute of Health and Wellbeing, University of Glasgow, 25 Bute Gardens, G12 8RS, Glasgow, UK; 2 Healthcare Improvement Scotland, 50 West Nile St, G1 2NP, Glasgow, UK; 3 Social Dimensions of Health Institute, University of Dundee, 11 Airlie Place, DD1 4HJ, Dundee, UK

**Keywords:** Patient reported outcome measures, Quality improvement, Chronic obstructive pulmonary disease, Low literacy, Learning disabilities

## Abstract

**Background:**

Patient reported outcome measures (PROMs) are self-report measures of health status increasingly promoted for use in healthcare quality improvement. However people with low literacy skills or learning disabilities may find PROMs hard to complete. Our study investigated stakeholder views on the accessibility and use of PROMs to develop suggestions for more inclusive practice.

**Methods:**

Taking PROMs recommended for chronic obstructive pulmonary disease (COPD) as an example, we conducted 8 interviews with people with low literacy skills and/or learning disabilities, and 4 focus groups with 20 health professionals and people with COPD. Discussions covered the format and delivery of PROMs using the EQ-5D and St George Respiratory Questionnaire as prompts. Thematic framework analysis focused on three main themes: *Accessibility*, *Ease of Use*, and *Contextual factors*.

**Results:**

*Accessibility* included issues concerning the questionnaire format, and suggestions for improvement included larger font sizes and more white space. *Ease of Use* included discussion about PROMs’ administration. While health professionals suggested PROMs could be completed in waiting rooms, patients preferred settings with more privacy and where they could access help from people they know. *Contextual Factors* included other challenges and wider issues associated with completing PROMs. While health professionals highlighted difficulties created by the system in managing patients with low literacy/learning disabilities, patient participants stressed that understanding the purpose of PROMs was important to reduce intimidation.

**Conclusions:**

Adjusting PROMs’ format, giving an explicit choice of where patients can complete them, and clearly conveying PROMs’ purpose and benefit to patients may help to prevent inequality when using PROMs in health services.

## Introduction

‘Patient Reported Outcome Measures’ (PROMs) are widely promoted as a means to monitor patients and to drive quality improvement in healthcare [1]. However most PROMs require literacy levels and cognitive abilities that can make them difficult for some people to use. This difficulty may in turn exclude them from completing PROMs and participating in PROMs’ subsequent uses.

This qualitative study investigates stakeholder views on PROMs, taking chronic obstructive pulmonary disease (COPD) as an example. In discussing PROM accessibility and usability issues with people with low literacy skills, learning disabilities and/or COPD, and health professionals, the findings provide insight into the potential to use PROMs more inclusively.

## Background

PROMs are questionnaires used to help understand the impact of healthcare on people’s health and quality of life. In the U.S., the Medicare Health Outcomes Survey is regularly administered to a sample of Medicare beneficiaries to drive quality improvement [2]. In England the Department of Health’s national PROMs programme helps to measure the outcome of varicose vein, hernia, and hip and knee replacement surgery. In this programme providers administer PROMs pre- and post-operatively to measure change in function and health related quality of life directly from patients [3]. The use of PROMs for quality improvement beyond acute procedures to long term conditions, including COPD, is imminent [1]. In Scotland one of the approaches highlighted in the quality strategy is to measure patient reported outcomes by using PROMs across the health service [4].

Before PROMs are put into practice they require rigorous development and testing which should involve the eventual users but in reality excludes certain groups. To ensure that PROMs are meaningful and relevant, people with the target conditions should help to decide which items to include, and participate in further validity testing [5]. But people with low literacy skills and people with learning disabilities are excluded from the PROMs development process [6]. As a result the PROMs may not be accessible to them; they may not understand the questions or response sets, decide not to complete it, or circle answers on PROMs at random. A study of those with suspected sleep apnoea using the Epworth Sleepiness Scale reported that while some participants were upfront about literacy issues, 33.3% of all first time users completed the form incorrectly or requested assistance from health professionals. Common errors included leaving questions blank and nonsense marking rather than on the scale [7].

When PROMs are put into practice, people with learning disabilities or low literacy skills might also struggle with an issue more subtle than accessibility – PROMs’ usability in clinical practice settings - what we refer to as PROMs’ ‘ease of use.’ People with learning disabilities or low literacy might find it more difficult to complete PROMs in some environments than in others. Fayers and Machin [8] suggest patients should ideally complete PROMs in waiting rooms and home completion is next best, but they do not suggest when particular settings may be more appropriate. Previous minimal guidance on administering PROMs also does not consider provisions for assisted self-response [1]. People with learning disabilities or low literacy may benefit from specific administration methods.

People with learning disabilities specifically have previously been shown to face exclusion due to inequity in standard clinical guideline development processes [9]. This group, along with people with low literacy skills, will also face exclusion from routine patient monitoring and broad quality improvement schemes based on PROMs data if they cannot use PROMs to accurately convey their perceived health, wellbeing and functioning. This issue is particularly pertinent in COPD because of its high prevalence in lower socio-economic and older groups where lower levels of literacy are also more common [10].

Previous initiatives have described the development of technology-driven tools, like visual touch screens in the place of paper-based questionnaires, which have shown promise in some low literacy populations [11,12]. While these may be helpful, they are not offered routinely or beyond selected populations. Despite there being 1.5 million people with learning disabilities in the UK [13] and one in five adults having low literacy skills [14], governments and NHS organisations are pressing ahead the PROMs agenda with relatively little consideration of how to include those who may find it difficult to complete the tools.

In this study, we investigate the views of people with learning disabilities and/or low literacy, health professionals, and people with COPD on the accessibility and ease of use of PROMs recommended for respiratory patients and in the NHS.

## Methods

This study uses two PROMs, the EQ-5D and the St George Respiratory Questionnaire, to gather participant views on PROMs’ accessibility and ease of use.

The EQ-5D is a generic PROM that was originally developed by a European collaboration as a brief self or interviewer administered questionnaire to be used alongside disease-specific measures as a common reference tool [15]. The items were derived from existing tools. The first part has five domains: anxiety/depression, mobility, pain, self care and usual activities of daily living. Respondents are asked to tick the option that best reflects how they feel. The second part is a 1–100 scale where participants are asked to draw a line to the number best assessing their health state ‘today.’ [16] In England, data collected using this measure are reported monthly for hip and knee replacement, varicose vein, and hernia surgical patients demonstrating the EQ-5D’s large scale feasibility [17].

The second PROM is the condition-specific St George Respiratory Questionnaire (SGRQ). Members of the public or patients were not consulted in the initial selection of items for this tool, but adults with asthma and airways disease were involved in determining further properties [18]. The first part of the measure focuses on respiratory symptoms over a time period and patients respond using a 5-point Likert scale. The second part focuses on the physical, social and psychological impact of the respiratory condition using true and false responses. The measure is meant to be self-administered with staff supervision [19]. The SGRQ has also been validated for phone administration [20]. It is mainly considered useful in pulmonary rehabilitation settings and research [16].

### Ethics

The research was approved by Stirling University and NHS ethics committees. All information sheets and consent forms were designed to be accessible to those with low literacy or learning disabilities and we obtained informed consent from all participants.

### Design

We used a descriptive qualitative study design. This included focus group discussions with health professionals and people with COPD, and individual interviews with people with low literacy skills and/or learning disabilities. We chose these different methods based on preliminary discussions about their suitability with professionals, support workers, and people with COPD.

### Recruitment

Convenience sampling was used to identify participants in four Scottish health board areas. People with COPD (without known low literacy or learning disabilities) were recruited through existing peer groups affiliated with a charity that focuses on chest conditions. We included participants with a known diagnosis of COPD. Health professionals who work with people with COPD were recruited through professional contacts in COPD services and national clinical networks. People with low literacy skills or learning disabilities were recruited by asking healthcare professionals in respiratory services and various community learning disabilities teams to invite clients they thought would be interested in participating in the study. We included participants who had known low literacy or learning disabilities, and were accessing community or clinical health services at the time of recruitment. Those interested gave permission for the lead researcher to contact them through telephone or mail to arrange an interview. Signed, informed consent was obtained in person with assistance from a support worker or carer when it was helpful for the participant.

### Topic Guide

We discussed our draft topic guide with a group of people with learning and communication difficulties and health professionals to ensure the wording, content, and format were appropriate. The final version of the topic guide covered the same topics in all three participant groups (people with COPD, people with low literacy/learning disabilities, and health professionals) and is shown in Table 1. After discussing form-filling in general we showed participants the EQ-5D and the SGRQ, and prompted them to comment on accessibility in relation to the formatting, style, colours, the addition of pictures, and anything else about the visual impression of the PROM. We then addressed ease of use issues such as location to complete the PROM and preferred help. In this way the guides covered literacy, accessibility and ease of use, and opened the discussion for any other issues relating to PROMs.

**Table 1:** Topic Guide Used for Interviews and Focus Groups

**Theme**	**Guiding Questions**
**Literacy Screening**	1. How often do you have someone help you read health information
	2. How confident are you filling out medical forms on your own
	3. How helpful is the written information that you receive
**Accessibility**	1. How does this PROM look?
	2. Is there anything that could make it look better?
	*Ex*. The instructions
	The size of the letters
	The way the letters look
	The headings
	Colour of paper and colour of writing
	Words used
	Length of sentences
	Placement of tick boxes
	3. Is there anything that could make it easier to complete?
**Ease of use**	1. Where would you prefer to complete this questionnaire? Why?
	*Ex*. Home
	Doctor’s room
	Waiting Room
	Private Room in hospital
	2. When would you prefer to complete this questionnaire? Why?
	*Ex*. When you arrive
	While seeing the doctor
	After seeing the doctor
	After you go home
	3. Would you prefer someone to help you complete this questionnaire? Who?
	*Ex*. Doctor
	Nurse
	Family member
	Friend
	No one

### Procedure

The data were collected in June 2011. Interviews with people with low literacy skills/learning disabilities took place in participants’ homes and focus groups with professionals and people with COPD took place in hospitals or community centres. All focus groups and interviews, were audio recorded and transcribed verbatim.

For five respondents the researcher used Talking Mats©, a picture-supported tool developed at Stirling University and used extensively in previous research with participants who might have difficulty communicating their views [21]. Each topic was presented on a card with an associated illustration. While discussing the topic the participant was asked to place the card on a mat to indicate his or her level of un/happiness or dis/agreement. This approach allowed participants to address topics consistently and systematically when an open discussion might have been too confusing. The interviewer took pictures of completed Talking Mats that were later mailed to participants to keep. All interview participants were given a nominal £10 gift token.

### Analysis

Interviews and focus group discussions were analysed using thematic framework analysis supported by Microsoft Excel 2007. As Ritchie, Spencer and O’Connor [22] describe, this type of analysis involves listing themes in columns across the top of a table and the associated transcripts in rows on the left side. The table is populated with transcript segments under each code. DJ developed and refined an initial coding frame based on the research questions. Each member of the research team applied the coding frame to one transcript to verify its accuracy and completeness and discrepancies were discussed and resolved in the team. Through ongoing discussion and scrutinizing the framework table, the initial codes were then combined into 3 larger themes. Similar themes emerged across the different participant groups indicating saturation of ideas so we did not conduct more interviews after the preliminary analysis.

## Results

We conducted two focus groups with people with COPD and two with health professionals. The focus groups had four to five participants. Two of the focus groups (one with people with COPD, and one with health professionals) only had female participants and the other two were mixed. The health professionals included allied health professionals, a consultant, a general practitioner, nurses, clinical care managers, and other roles associated with COPD care. All had experience administering PROMs or a particular interest in the measures. The participants with COPD were all above age 50 and had been participating in a charity-sponsored COPD peer group for at least five years.

We also interviewed eight adults who were judged by a health professional to have low literacy skills or who had been diagnosed and in receipt of support for any form of learning disability. The participants included six males and two females who resided in various towns across four health boards in Scotland. Two participants had been diagnosed with COPD, and were recruited through COPD services, and the remaining were recruited through community nursing and learning disabilities focused services. All participants were accessing local community or health services for various conditions. One participant with low literacy had recently learned to read through an adult reading programme. The other participants had varying degrees of both low literacy and learning disabilities. For three participants, these limitations resulted in them needing assistance from a support person to respond to interview questions; one of these participants resided in a care home.

We use the following abbreviations to present the results:

People with low literacy skills or learning disabilities: LLLD

People with COPD: PWCa and PWCb (2 focus groups)

Health professionals: HPa and HPb (2 focus groups)

### Accessibility

Accessibility issues included discussion about the actual PROM questionnaire rather than its administration although both issues are related.

#### ***Complexity***

The EQ-5D was seen as largely straightforward while participants thought the SGRQ was complicated at first glance. The appearance of the EQ-5D made it seem easy but patients and professionals reported that they did not like the EQ-5D visual analogue scale (VAS):

“This is too confusing, [patients] wouldn’t understand that would they…the word imaginable, worst imaginable, I think you have to use the words good or bad,” LLLD 6 support worker.

“I’m lost already. [Reads question out loud more slowly],” PWCa 2.

Participants favoured a simple tick or a 1–10 scale in line with other scales they had used or administered to the EQ-5D VAS. In contrast to initial views of the EQ-5D, some participants responded negatively to the SGRQ in terms of it length and format, “*if I got that*, *I would probably end up putting it in the bin*,”(LLLD 2). But the form was largely said to be understandable once participants with COPD and those with learning disabilities (who could read) read it slowly. Professionals reported that an example of a tick box would help to guide PROM completion.

#### ***Formatting***

The formatting affected the perceived accessibility of the PROMs and was associated with the PROM’s complexity. Questions, response options, headings, and tick boxes that were aligned made the PROMs seem easier. In this light, participants praised the EQ5D but found the SGRQ, “*quite messy looking*…*nothing seems to* [*follow*] *in a kind of order*,”(HPa 3). Participants said that a font size of at least 14 pt and larger tick boxes would enhance the accessibility of both PROMs. Bold, prominent headings, as on the EQ-5D, were also favoured.

#### ***Wording***

There were specific issues with the wording of items in the PROMs. Participants with COPD and health professionals challenged the EQ-5D’s emphasis on a person’s health state ‘*today*’:

“I don’t even know how I would advise [a client] to answer it, is it how bad they feel their condition is or how bad they feel today or…” HPb 3.

“[It] depends when they give you the form to fill in…It’s like you have a good day and you have a bad day, so is it mobility on your good day or mobility on your worst day?” PWCa 3.

All three groups suggested consistency in question types, wording, and the style of response options would make the PROM easier. Two particular question styles were difficult for participants to answer. First those with two or more aspects to consider:

“Yeah, [the SGRQ] is alright but it’s a bit long winded…I mean, one, two, three, four, five, six different things there that, you know, it’s…like, gardening is weeding, you know, so that’s one [referring to section six of the SGRQ]” LLLD 7.

Second, questions requiring recall were particularly difficult, e.g. remembering symptoms 3 months ago as required by the SGRQ.

### Ease of use

We classified issues related to the logistics and administration of PROMs under this theme, for example, the setting and desired assistance to complete PROMs.

#### ***Setting***

People with LLLD and/or COPD generally reported that completing a PROM at home was easiest. Their main concerns were ensuring confidentiality, having enough time to complete it, and being in a distraction-free environment. In line with these concerns, completing a PROM in a waiting room was unpopular:

“[Because] if it’s to be personal and you’re filling it in, in a waiting room, other people could see it…” LLLD 2.

“… especially [if] there are kids there you see… I like it quiet,” LLLD 3.

Contrary to these views, some health professionals said that time spent waiting for appointments could be used to complete PROMs. They also felt that if given for home completion patients may “*sit at home and stew over it* [*whereas their*] *spontaneous first answer is usually the right one*,”(HPa 1).

#### ***Assistance***

Both people with LLLD and participants with COPD reported they would like someone they trust to help them with a PROM. For example most people regardless of literacy level already had someone to help with general form filling, “*A family member*,”(PWCa 1), “*I*’*ve got a carer who comes in*,”(PWCa 2)“*My daughter*,”(PWCa 3) “*My sister*-*in*-*law*, *my sister*,”(PWCa 4). Participants associated the assistance available to them with the location where they complete PROMs. Professionals suggested if PROMs were completed in waiting rooms, patients could tell the receptionist or bring the PROM to the appointment if they struggled. But people with LLLD or COPD reported that in completing PROMs at home they could access their usual and preferred help.

People who know the patients well were viewed by patients and health professionals alike as being the preferred choice in assisting with the completion of forms. However they also pointed out that interference can result the patient’s voice getting lost.

#### ***Time***

There were mutual concerns about having enough time to complete PROMs during a consultation:

“[You] don’t obviously have time to fill out the [Clinical Respiratory Questionnaire for example], because it is quite lengthy…so hence the reason why you would maybe send it home with them,” HPb 1.

“Doctor’s appointments are, what, ten minutes, you wouldn’t have the time,” LLLD 7.

Despite uncertainty about completing PROMs with health professionals because of a lack of time, both people with LLLD and participants with COPD preferred to have assistance over attempting the form alone.

### Contextual factors

Contextual factors included challenges not highlighted in previous sections and wider issues associated with completing PROMs, low literacy, and learning disabilities.

#### ***Embarrassment and Lack of Confidence***

Health professionals and patient participants each described embarrassment in the face of low literacy. Some people with COPD admitted they may not ask for help even if they do not understand a questionnaire they are meant to complete. Professionals reported that often patients will say that they forgot their glasses or other excuses to hide low literacy. In turn professionals do not like to confront patients with suspected low literacy for fear that the patient will not return to the clinic if labelled as such on their record. Professionals expressed frustrations with the system because of the constraints to spending the needed extra time with people with learning disabilities and the inability to recognize literacy issues:

“[A patient] who I was completely losing the battle with, it was only when his GP phoned me up and said, you know, you do realise he can’t read…that finally we were able to try to get some headway into things and I started drawing lots of pictures but, you know, guys who are 32 and work as tree surgeons with chainsaws, you wouldn’t think they would be illiterate,” HPa 1.

People with LLLD or COPD also highlighted a lack of confidence in completing forms on their own regardless of their literacy level. For some the fear to complete forms by themselves was compounded with bad experiences:

“I feel bad about filling in forms on my own… after the experience that I’ve had years ago, by filling in a form for disability living allowance [national benefits programme for people with learning disabilities]. I got turned down for that and I knew that I was entitled to it. So I took it to my support worker and he filled it in for me. And as soon as he filled it in, I got disability money, [no] problem. So it must have been something that I done to fill it in wrong,” LLLD 2.

#### ***Lack of clarity about the benefit of PROMs***

All stakeholders emphasized the need to understand and see the benefit of PROMs. Patients spoke about past experiences where they did not know the purpose of forms, making them feel suspicious or intimidated, and frustrating experiences completing forms where they had never seen results. Specifically, participants were keen to use PROMs if they knew the measures would help them to express their thoughts to their doctor, see improvements in their condition, and help others. Health professionals had a similar wish to understand the purpose and benefit of PROMs highlighting that they would like to use them as tools to monitor patients, better understand their symptoms, and determine practical action to improve patient health.

## Discussion

This study is the first of its kind to highlight the challenges associated with using PROMs with populations who may require specific assistance, such as those with low literacy and/or learning disabilities. According to our findings the EQ-5D and the SGRQ, in their current form, may have limited accessibility for these groups. Participants advocated for changes in the format (e.g. larger font sizes) and mode of administration (e.g. assistance with completion) of PROMs. The findings are also pertinent to others who find it difficult to engage with written material due to impairments of memory, attention, or comprehension (e.g. some people with dementia, acquired brain injuries). The actions taken to support people with low literacy skills or learning disabilities may have wider benefits beyond these groups.

Leverage-saliency theory of survey participation helps to frame the discussion about how the attributes of a questionnaire impact people’s ability and desire to respond in various ways.[23] ‘Salient attributes’ are the features of the survey that a person considers important in their decision on whether to complete the questionnaire. ‘Leverage’ is the weight the person gives to a salient feature. For example, someone may consider font size salient, and it may carry high positive leverage, while s/he does not like the topic of the questionnaire so it carries negative leverage. If the font size is big enough to make the tool easy to use, it can overcome a person’s negative feelings about the survey topic, and s/he will be more likely to complete the questionnaire.

PROM formatting and their administration were salient attributes for participants and could be addressed to improve participants’ ability to complete PROMs. The format of current paper-based questionnaires was considered feasible with modifications that are typical of information given to people with learning disabilities (such as large font size and consistency) [24]. In contrast to other studies and initiatives [11,12] our participants did not suggest technology-supported solutions to PROMs administration but this might be because it was not explicitly offered as an option. While formatting modifications were important, the way PROMs were administered was seen as even more significant and thus also carry more leverage. Completing PROMs at home was seen to make PROM completion easier because in this setting people could access their preferred source of support. Guidance in the past has focused on waiting rooms [8], which may not work for all participants. Patient participants, regardless of reading ability, gave this issue considerably higher salience than health professional participants. While one of our professional participants suggested sending PROMs home would result in over-thought answers, it was exactly this opportunity to have the time to think and concentrate on a PROM that patient participants felt was necessary to complete the PROM accurately. Without the time to focus, which often is not provided in clinical settings, they may feel frustrated or intimidated to complete it without assistance.

The perception of form-filling based on past experience was also salient but carried negative leverage that could potentially be overcome through a clear understanding of the purpose of PROMs. Our findings suggest that previous negative experiences with form filling lowered some participants’ confidence to complete forms, and that participants were concerned that PROMs were ‘just another form’ for which they would never see feedback. These perceptions carried negative leverage as they made participants less likely to want to complete PROMs. But participants spoke positively when they understood PROMs as beneficial to help express their perceived health and wellbeing to professionals and help others with this information. This perception suggests that the purpose and use of PROMs is also salient to users. In addition to matching the formatting and administration of PROMs with the respondents’ assistance requirements, clearly conveying the benefits of the tools to participants may foster their ability to complete them, and overcome the negative leverage carried by the negative perception of form-filling in general. Health professionals have also repeatedly highlighted the need to see a purpose and benefit to clinical practice before they consider PROMs worth administering [1], and it is equally important to feedback the results directly to patients.

Tailoring PROMs administration, as our findings suggest may be helpful, is potentially controversial because the purist view of PROMs is that they should be delivered in exactly the same format that they were developed to ensure validity and comparability. For example, the authors of the SGRQ state that the questionnaire should be completed without any assistance because of the potential bias introduced to the patients’ responses [19]. However, Harniss et al. [25] point out that accommodations, such as the formatting and administration methods our participants suggested, can also reduce the bias associated with excluding a large number of people from using PROMs. Pragmatically, practitioners may need to balance the requirement to use PROMs exactly how they were developed with the need to ensure accessibility and full use in practice by making some amendments to formatting and administration. In some cases researchers have validated alternative methods for PROMs (e.g. phone administration of the SGRQ [20]) or guidelines have suggested that a clinician should be on hand while a patient is completing a PROM to answer questions [19]. Even under these circumstances, the degree and type of assistance and tailoring that is required will vary and typically go beyond solely having a clinician on hand to answer questions. We cannot propose a definite solution to the sometimes contradictory theoretical and practical nature of PROMs. However, considering this issue using leverage-saliency theory suggests that easy read modifications and assistance to complete PROMs could increase the likelihood that people with low literacy skills or learning disabilities will be able to use PROMs as they are both highly salient.

There were some limitations that may affect the applicability of our findings beyond our study. Our sample was small and there is limited work on this issue in the PROMs literature to ground our findings. However, we did reach topical saturation as similar themes emerged from various participants after the preliminary analysis. Our findings regarding formatting are also consistent with existing guidance for creating accessible information for people with learning disabilities [24]. As we recruited professionals through contacts and involved people who were already accessing support from the third sector or from community health services, the participants may have already had an interest in research and PROMs. Finally, sometimes it was difficult to decide whether a participant was influenced by her/his support worker, or was responding to please without full comprehension of the question.

## Conclusion

There are three main considerations to support the inclusion of people with low literacy skills or learning disabilities in initiatives based on PROMs. First is to format PROMs to adhere to existing accessibility principles including large font sizes and ample white space. The second is to allow flexibility so that patients can choose where to complete PROMs and request assistance from people they know or from professionals. Finally, clearly conveying the purpose and benefit of PROMs to both patients and professionals may also support inclusivity by reducing intimidation and frustration caused by form filling in general. These principles may help to prevent the exclusion of people with low literacy skills or learning disabilities from using PROMs and participating in subsequent patient monitoring and health service quality improvement processes.

## Abbreviations

PROM: Patient Reported Outcome Measure; COPD: Chronic Obstructive Pulmonary Disease; EQ5D: EuroQol-5D; SGRQ: St George Respiratory Questionnaire.

## Competing interests

The authors declare that they have no competing interests.

## Author’s contributions

DJ carried out the interviews and focus groups, and drafted the manuscript. SW led the study’s conception and its design though all authors contributed. TK and SW participated in designing the methods, analysis and coding frame, and revised several drafts. KR participated in coordinating the study, refining the coding frame and revising several drafts. All authors read and approved the final manuscript.

## Pre-publication history

The pre-publication history for this paper can be accessed here:

http://www.biomedcentral.com/1472-6963/12/431/prepub
